# Supplementary material for: Spray-Dried Formulation of Epicertin, a Recombinant Cholera Toxin B Subunit Variant That Induces Mucosal Healing
Source: Pharmaceutics. 2021 Apr 18;13(4):576. doi: 10.3390/pharmaceutics13040576 (PMC8073836; doi:10.3390/pharmaceutics13040576)
Supplement: Supplementary file 1 [file pharmaceutics-13-00576-s001.zip › pharmaceutics-1169604-supplementary.pdf]

pharmaceutics

Supplementary Materials: Spray Dried Formulation of Epicertin, a Recombinant Cholera Toxin B Subunit Variant that Induces Mucosal Healing

Micaela A. Reeves, Joshua M. Royal, David A. Morris, Jessica M. Jurkiewicz, Nobuyuki Matoba and Krystal T. Hamorsky

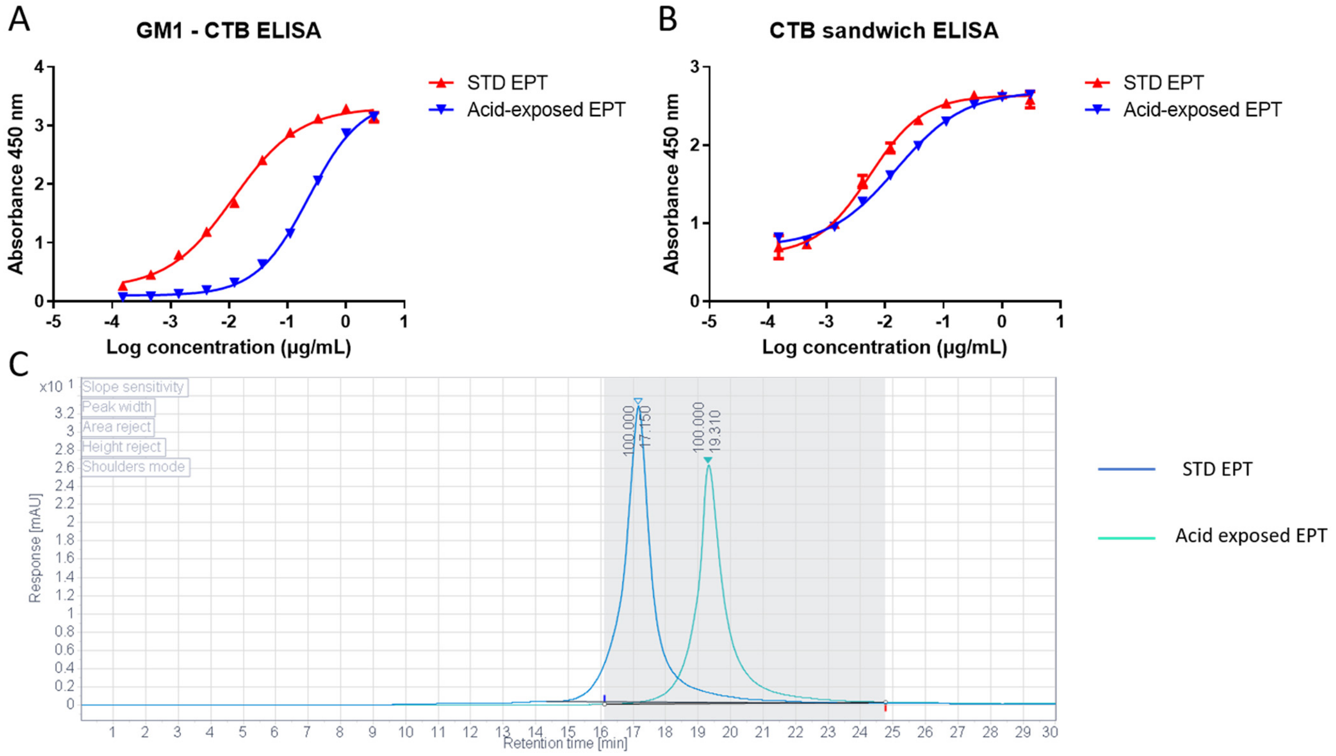

Figure S1. Evidence for EPT degradation at pH 1.0, These results demonstrate the need for a pH-dependent oral formulation of EPT. Top left: GM1-CTB ELISA. STD EPT in pentamer has a much higher EC50 to GM1 than acid-exposed EPT which contains EPT in monomer form. Top right: CTB sandwich ELISA. STD EPT in pentamer form and acid exposed EPT monomer form can be detected in a similar manner demonstrating capacity to detect both GM1-binding pentamer and disassembled CTB molecular species. Bottom: Size Exclusion (SEC)-HPLC. STD EPT elutes in pentamer form at a 17.1 min retention time whereas acid exposed EPT elutes in monomer form at 19.3 min, whereby demonstrating acid dissociates pentamer EPT into monomer EPT.

Publisher's Note: MDPI stays neutral with regard to jurisdictional claims in published maps and institutional affiliations.

Copyright: © 2020 by the authors. Submitted for possible open access publication under the terms and conditions of the Creative Commons Attribution (CC BY) license (http://creativecommons.org/licenses/by/4.0/).
